# CXC Ligand 5 cytokine serum level in women with polycystic ovary syndrome and normal body mass index: A case-control study

**Published:** 2017-10

**Authors:** Marzieh Zohrabi, Elham Rahmani, Niloofar Motamed, Samad Akbarzadeh

**Affiliations:** 1 *The Persian Gulf Tropical Medicine Research Center, Bushehr University of Medical Sciences, Bushehr, Iran.*; 2 *Department of Obstetrics and Gynecology, Faculty of Medicine, Bushehr University of Medical Sciences, Bushehr, Iran.*; 3 *Department of Community Medicine, Faculty of Medicine, Bushehr University of Medical Sciences, Bushehr, Iran.*; 4 *Department of Biochemistry, Faculty of Medicine, Bushehr University of Medical Sciences, Bushehr, Iran.*

**Keywords:** Polycystic ovary syndrome, Insulin resistance, CXCL5 cytokine, Hyperinsulinemia, Hyperandrogenism

## Abstract

**Background:**

Polycystic ovary syndrome (PCOS) is the most common endocrine disease and associated with insulin resistance. CXC Ligand 5 (CXCL5) is a new cytokine which is secreted from white adipose tissue during obesity and by blocking insulin signaling pathway inhibits the activity of insulin and promotes insulin resistance.

**Objective:**

The aim of this study was to assess serum level of CXCL5 in PCOS women with normal body mass index.

**Materials and Methods:**

In this case-control study, 30 PCOS women with normal body mass index as the case group and 30 non-PCOS women as the controls were enrolled. Serum levels of CXCL5, insulin and other hormones factors related with PCOS were measured by ELISA method, also the biochemical parameters were measured by autoanalyzer.

**Results:**

Significant increases in serum insulin concentration, homeostasis model assessments of insulin resistance, luteinizing hormone, luteinizing hormone/follicle-stimulating hormone, fasting blood sugar, testosterone, and prolactin were observed in the case group compared to the controls. were in the serum level of CXCL5, cholesterol, low-density lipoprotein-cholesterol, high-density lipoprotein-cholesterol,dehydroepiandrosterone-sulfate, creatinine, and homeostasis model assessment of beta cell function between these two groups.

**Conclusion:**

In this study, no significant change was observed in serum concentrations of CXCL5 in PCOS women with normal BMI.

## Introduction

Polycystic ovary syndrome (PCOS) is the most common endocrine disease in women (1). The prevalence of PCOS in the world is one in every 15 women and affects 4-8% of women in the reproductive age (2, 3). According to the diagnostic criteria of Rotterdam in 2003, PCOS women should have at least two of the three criteria such as Oligo or anovulation, clinical hyperandrogenism and show a polycystic ovary on ultrasound (4). This disease is characterized by insulin resistance, pancreas beta cell dysfunction, impaired glucose tolerance, diabetes type II, dyslipidemia, and visceral obesity (5, 6). 

Previous studies showed that insulin resistance in 50% of women with PCOS is related to excessive phosphorylation of serine in insulin receptor by the serine/threonine kinas enzyme (7, 8). This process can inhibit autophosphorylation of tyrosine in insulin receptors, without any effect on insulin binding leading to insulin resistance in PCOS patients (7). This enzyme also increases serine phosphorylation of the P450c17 enzyme since it is a key enzyme for controlling androgen biosynthesis that can lead to increased production of androgens (9). As a consequence, insulin resistance and hyperandrogenism can be seen in PCOS women (10, 11). CXC Ligand 5 (CXCL5) is a new cytokine which inhibits the activity of insulin in muscles and promotes insulin resistance and secreted from white adipose tissue during obesity (12). This cytokine is produced by different cells such as monocytes, neutrophils, epithelial cells, fibroblasts and smooth muscle cells (13). Circulating CXCL5 is highly increased during obesity in both mice and humans (14, 15). CXCL5 by blocking insulin signaling pathway through activating the pathway of Jak2/STAT5/SOCS2, plays an important role in the development of insulin resistance (15). Following injection of anti-CXCL5 antibody or CXCR2 antagonist as CXCL5 receptor, in obese mice improves insulin resistance and decreases fasting glucose levels (16). 

Due to the positive association of CXCL5 with insulin resistance and the important role of insulin resistance in the pathogenesis of PCOS and since this cytokine has been studied in obese people so far, we have decided to evaluation this cytokine in PCOS women with normal body mass index.

## Materials and methods

In this case-control study, 30 PCOS women with normal body mass index (case group) and 30 non- PCOS women with normal body mass index (control group) referred to the Abolfazle Clinic, dependent to Bushehr university of medical scinces, Iran from March 2015 to September 2016 were enrolled. The sample size was obtained with a confidence level of 95% and a test power of 80%.

**Inclusion and exclusion criteria**

Inclusion criteria in the case group were, according to the diagnostic criteria of Rotterdam in 2003, PCOS women should have at least two of the three criteria covering: Oligo or anovulation, clinical hyperandrogenism and show a polycystic ovary on ultrasound (4). 

Control group were selected from women with normal menstruation cycles, normal pelvic ultrasound, without acne, as well as hirsutism and infertility.

Both groups had mean body mass index (BMI) of 25 kg/m2) and age of 40 yr. All women with thyroid disorders, tumors, cardiovascular diseases, diabetes mellitus, hypertension, renal disorders, and taking medications such as contraceptive pills, glucocorticoids, or hypertension, estrogenic and androgenic drugs in the last three months were excluded.

**Sampling and measurement of parameters**

Blood samples from the vein of the right-arm were obtained in the follicular phase (1st to 4th day of menstruation cycle) after 12 h of overnight fasting. Serum was obtained after centrifugation (3000 rpm for 15 min at 4^o^C), and then the serum was frozen at -80^o^C. Anthropometric parameters including age, weight, height, body mass index and waist to hip ratio were measured using questionnaires, scales and meters. Biochemical parameters including fasting blood sugar (FBS), Triglyceride (TG), Cholesterol, High-density lipoprotein cholesterol (HDL-C) and creatinine (Cr) were measured by autoanalyzer Selectra E (Vital scientific N.V, The Netherlands). Reliable enzymatic techniques were used for measurement of FBS, TG, Cholesterol, Cr, Testosterone, Dehydroepiandrosterone-sulfate) DHEAS) and Prolactin (Pars Azmoon-co, Iran). 

The HDL-C levels were measured after precipitation with magnesium chloride by enzymatic techniques (Pars Azmoon-co, Iran) and the low-density lipoprotein cholesterol (LDL-C) levels were calculated by using the Friedwald formula (17). The serum levels of insulin and CXCL5 (Crystal Day, China) were measured by ELISA technique (Dynex Technologies 2cxb3510, USA). Homeostasis model assessment of insulin resistance (HOMA-IR) and homeostasis model assessment of beta-cell function (HOMA-B) were calculated by using the following formula (18, 19).

$\mathrm{HOMA.IR}=\frac{\mathrm{Fasting Insulin}\left(\mu \frac{\mathrm{IU}}{\mathrm{ml}}\right)\times \mathrm{Fasti}\mathrm{ng Glucose}(\frac{\mathrm{mg}}{\mathrm{dl}})}{405}$

$\mathrm{HOMA.B}=\frac{\mathrm{Fasting Insulin}(\mu \frac{\mathrm{IU}}{\mathrm{ml}})\times 360)}{\mathrm{Fasti}\mathrm{ngGlucose}\left(\frac{\mathrm{mg}}{\mathrm{dl}}\right)-63}$

**Ethical consideration**

The research protocol was approved by the Ethics Committee of Bushehr University (Id number: “IR.BPUMS.AC.REC.1394.100). A written informed consent was obtained from all participants.

**Statistical analysis**

We used Statistical Package for the Social Sciences (SPSS, version 16.0, SPSS Inc, Chicago, Illinois, USA). Independent samples t-test and Chi-square test were used to compare the quantitative variables in the two groups and all the results expressed as mean±SD. The single sample Kolmogrov-Smirnov test was used to estimate the variables’ distribution characteristics. p<0.05 was considered significant.

## Results

There were no significant differences in the demographic characteristics such as weight, height, age, body mass index and the waist- hip ratio between case and control groups (Table I). There were significant increases in serum level of Luteinizing hormone (LH) (p=0.004), LH/Follicle-stimulating hormone (FSH) (p=0.006), testosterone (p=0.02), and prolactin (p=0.04), in the case group compared with the controls (Table II). A significant increase was observed in parameters such as FBS (p=0.01), serum insulin level (p=0.04), and HOMA-IR (p=0.04) between two groups. Although, serum CXCL5 level were obtained higher in the case group compared to the controls but this difference was not significant (Table II). Also, there were no significant variations in the amount of TG, Chol, HDL-C, LDL-C, FSH, HOMA-B Dehydroepiandrosterone-sulfate, and Cr in both groups (Table II).

**Table I.** Demographic characteristics of the two study groups (n=30)

**Characteristic **	**Control group **	**Case group**	**p-value**
Age (year)	28.91 ± 8.1	25.85 ± 5.90	0.12
Weight (Kg)	62.47 ± 15.18	66.74 ± 12.41	0.28
Height (cm)	161.17 ± 4.71	159.52 ± 6.37	0.30
BMI (Kg/m^2^)	24.02 ± 5.60	24.91 ± 3.63	0.40
waist-hip ratio	0.8 ± 0.07	0.82± 0.06	0.27

All data presented as Mean ± SD.

Independent samples t test

Chi-square test

BMI: body mass index

**Table II:** Serum concentrations of variables in patient and control groups (n=30

**Characteristic**	**Control group **	**Case group **	**p- value**
CXCL5 (ng/ml)	378.77 ± 340.57	473.75 ± 436.28	0.30
Insulin (μIU/ml)	8.76 ± 7.63	16.42 ± 17.01	0.04
HOMA-IR	2.06 ± 2.04	4.11 ± 4.29	0.04
HOMA-B	109.50 ± 71.75	179.17 ± 218.82	0.10
FBS (mg/dl)	91.29 ± 8.53	98.71 ± 11.44	0.01
Triglyceride (mg/dl)	86.79 ± 50.23	107.04 ± 45.03	0.13
Cholesterol (mg/dl)	167.83 ± 29.18	175.07 ± 23.32	0.30
HDL-C (mg/dl)	49.25 ± 12.59	47.92 ± 10.08	0.67
LDL-C (mg/dl)	100.96 ± 26.55	99.60 ± 25.72	0.62
LH (mIu/ml)	5.54 ± 2.84	9.46 ± 5.6	0.004
FSH (mIu/ml)	6.28 ± 2.13	5.24 ± 2.35	0.10
LH/FSH	1.06 ± 0.84	2.27 ± 1.92	0.006
Testosterone (ng/ml)	0.71 ± 0.26	0.99 ± 0.52	0.02
DHEAS (µg/dL)	1.83 ± 0.78	1.99 ± 0.87	0.40
Prolactin (ng/mL)	15.00 ± 8.84	21.23± 11.94	0.04
Creatinine (mg/dl)	0.80 ± 0.16	0.81 ± 0.13	0.70

Data presented as mean±SD. Independent samples t-test, Chi square test, single sample Kolmogrov-Smirnov test.

CXCL5: CXC Ligand 5

HOMA-IR: Homeostasis model assessments of insulin resistance.

FBS: Fasting blood sugar

HOMA-B: Homeostasis model assessments of B cell function

HDL-C: High-density lipoprotein cholesterol

LDL-C: Low-density lipoprotein cholesterol

LH: Luteinizing hormone

FSH: Follicle stimulation hormone

DHEAS: Dehydroepiandrosterone- sufate

## Discussion

Our results showed the significant increases in serum level of insulin and HOMA-IR in PCOS women compared with the controls. In accordance with these findings, Tun and colleagues reported, a significant increase in HOMA-IR level in PCOS women (20). Also, we showed a significant increase in FBS (p =0.01) in PCOS women. In fact, it can be stated that insulin sensitivity falls and insulin resistance is dominant for hyperinsulinemia in PCOS women. Layegh and coworkers observed in their study that insulin resistance increases in obese (80.3%) and non-obese PCOS (72.2%) women (21). In our study, a significant increase was observed in testosterone level (p=0.02) in the PCOS group compared to the control group. Chen and coworkers showed that intra-ovarian hyperandrogenism is one of the determining factors of follicular arrest in PCOS women. Also, it was determined that Dihydrotestosterone suppresses the FSH activity in granulosa cells of rats, increases accumulation of cells in Gap2/Mitosis phases and induces production of a number of large arrested follicles. This condition is similar to polycystic ovaries for women with PCOS (22). Sharquie and colleagues reported that free testosterone level can be used as the most sensitive biochemical marker in the diagnosis of PCOS (23). 

Also, we found the significant increase in the level of serum insulin, LH, and LH/FSH ratio. Azziz and colleagues showed that elevation in the serum levels of LH and insulin increase production of ovarian androgens synergistically (24). Insulin resistance stimulates insulin release, reduces sex hormone-binding globulin production and increases free testosterone (24, 25). Hyperandrogenism and hyperinsulinemia can lead to the development of ovarian follicles that can cause infertility in PCOS women (24). In this study, a significant increase was seen in prolactin level (p=0.04) in PCOS women compared with the control group. The similar results was achieved by Kumar and coworkers (26). Nizam and colleagues showed that increase in prolactin secretion is a major cause of sub-fertility (27). Therefore, treatment with drugs that lowered prolactin levels resulted in pregnancy for 24% of the infertile women (27-30). In a study, Kalsum and Jalali found hyperprolactinemia in 69.51% of infertile women (31). The results of this study showed an increase in CXCL5 level in PCOS group compared to the control group but this increase was not significant statistically; probably it can be attributed to low BMI of PCOS women in this study. Previous studies have shown that serum level of CXCL5 is highly increased during obesity in both mice and humans (12, 14, 32). 

Chavey and colleagues reported, in a study, a significant increase in serum level of CXCL5 in obese French people compared to lean French people (12). Also, they showed a significant positive correlation between serum level of CXCL5 and body weight in the French population and reported that inhibition of CXCL5 secretion in obese people can reduce the risk of developing obesity-related pathogenesis. So, inhibition of CXCL5 signaling can be used as a therapeutic modality for metabolic syndrome (12). On the other hand, Layegh and coworkers while assessing endocrine-metabolic disorders and insulin resistance in obese PCOS women (BMI >25), and non-obese ones (BMI <25), stated that metabolic abnormalities in obese PCOS women are more common than non-obese PCOS women. As, in 39.4% of obese PCOS women, metabolic syndrome was observed while no case of metabolic syndrome was seen in non-obese PCOS women (21). Thus, the serum level of CXCL5 increases with weight gain and obesity. It can be stated that probably CXCL5 in obese PCOS women can be effective in causing metabolic disorders associated with PCOS.

## Conclusion

In this study, no significant change was observed in serum concentrations of CXCL5 in PCOS women with normal BMI.
